# Evolution of Litter Size: Proximate and Ultimate Mechanisms

**DOI:** 10.1093/icb/icae052

**Published:** 2024-05-27

**Authors:** Kathryn Wilsterman, Anna Isabel Bautista, Chloe E Butler, Makenna Y Juergens, Ashley M Larson

**Affiliations:** Department of Biology, Colorado State University, Fort Collins, CO, 80521, USA; Department of Biology, Colorado State University, Fort Collins, CO, 80521, USA; Department of Biology, Colorado State University, Fort Collins, CO, 80521, USA; Department of Biology, Colorado State University, Fort Collins, CO, 80521, USA; Department of Biology, Colorado State University, Fort Collins, CO, 80521, USA

## Abstract

Relative reproductive success and failure are the ultimate determinants of Darwinian fitness. As such, reproductive traits and variations therein have an immediate and considerable impact on the evolutionary trajectory of lineages. Historically, significant attention has been paid to the ecological and evolutionary processes (ultimate factors) that shape the diversity and canalization of reproductive traits within groups to better our understanding of organismal diversity and population or species resilience. In contrast, the physiological systems that mediate variation within and among species (i.e., the proximate factors) in reproductive traits remain a significant black box. To date, there is comparatively little information about how proximate mechanisms constrain or promote evolutionary potential in reproductive traits. In this mini-review, we focus on litter size in Eutherian mammals as a trait with relatively well-defined diversity (litter sizes are well-described both within and across species) and for which some genetic determinants have been identified. We discuss both the ultimate and potential proximate determinants of litter size with special attention to the breadth of physiological traits that may act as “toggle” switches for evolution of litter size. We close with a brief discussion of the role that physiological plasticity may play in the evolution of litter size and lay out several forward-looking areas for future research.

## Introduction

A primary goal of the fields of ecological, evolutionary, and comparative physiology is to understand how physiology and physiological trade-offs influence organismal fitness in ways that impact a lineage’s capacity to persist through time or space. Reproduction is essential for species persistence; moreover, differential fitness (variation in the reproductive success of individuals) is ultimately necessary for evolution and adaptation. However, the reproductive physiologies that underlie species or lineage evolution and adaptation are relatively poorly understood, even in model systems, and especially when contrasted with survival-related traits (see Box 1). This historical focus has led to a large gap in understanding about how reproductive physiology contributes to local adaptation and the potential for species to invade or persist in changing environments.

The gap in knowledge is particularly notable in mammals. Although reproductive traits are well-known to vary across and within species, we know very little about the physiology that underlies that variation in all but a few (often exemplary) species. Even less is usually known about the *evolutionary* history of variation in reproductive traits within lineages. At least some of this lack of physiological insight is a product of the historical bias that female reproductive physiology is generally passive (see Hayssen and Orr 2017; Orr et al. 2020 for further context). These ideas are now broadly accepted as inaccurate; however, the literature bias remains and likely contributes to the relative dearth of general understanding about female traits that are likely explanatory candidates for local adaptation in reproductive traits.

In this manuscript, we aim to promote interest and understanding related to the evolutionary physiology underlying reproductive traits by focusing on the number of offspring per litter (i.e., litter size). Litter size is a tractable and timely trait to consider because (1) variation in litter size is demonstrably consequential for fitness, (2) natural variation both within and across species is well-documented, and (3) the reproductive physiology underlying the progression of development from ova to offspring is well understood in biomedical and agricultural models. It is arguably striking then that natural variation within and among mammalian lineages (reported litter sizes range from 1 to 30 offspring across species, Hayssen and Orr 2017) has not garnered more attention from evolutionary physiology or genomics.

Box 1: **Genotype-to-phenotype links for survival versus reproductive traits in a model rodent system.**

Deer mice (*Peromyscus maniculatus*) are a model system for evolutionary biology, in large part because they are extremely broadly distributed across North America, display local adaptation across North American habitats, and are amenable to lab experiments (Bedford and Hoekstra 2015). Local adaptation in reproductive function was first observed and documented through experimental approaches over 30 years ago, though these studies focused near-exclusively on male traits (e.g., Demas et al. 1996; Demas and Nelson 1998a, 1998b; Prendergast et al. 2001). Much more recently, a series of museum collection-based studies have documented ecologically linked variation in reproductive seasonality and litter size (pup number per litter) within deer mice (*Peromyscus maniculatus*; McLean et al. 2019, 2022; McLean and Guralnick 2021), and many of these population-level differences in gestational traits persist in common-garden, laboratory environments (Wilsterman and Cunningham 2022). Nonetheless, the biological determinants of natural variation in reproductive traits, from the genome to physiology, remain largely undetermined (but see Wilsterman et al. 2023). This is in striking contrast to the abundance of links from the genome to physiology and survival-linked traits in this model system (e.g., Linnen et al. 2009; Schwimmer and Haim 2009; Scott et al. 2018; Barrett et al. 2019; Hager and Hoekstra 2021; Rocha et al. 2021; Storz 2021; Hager et al. 2022; Williams and Tieleman 2005).

We first set the scene by briefly summarizing the large and well-established literature that addresses ecological determinants of litter size as an important context for understanding the ultimate determinants. We then introduce the proximate determinants of litter size in Eutherian mammals, referring here to the physiology that can dynamically influence the number of offspring produced in a single gestational bout. Throughout, we highlight examples where the proximate (physiological) determinants of litter size are known, though these are largely sourced from domesticated mammals. As such, we also discuss the limitations of drawing generalizable patterns from domestic strains, particularly as it relates to the physiological determinants of litter size. Finally, we briefly address physiological plasticity and potential trade-offs that may influence the extent to which litter size can evolve within populations in response to selective pressure, and we outline some outstanding challenges in investigating litter size evolution.

## Ultimate determinants of litter size

A plethora of theoretical frameworks have been proposed over many decades to explain the ultimate determinants of litter or clutch sizes within and among species. Many of these ultimate frameworks were developed using clutch size in birds as the focal model, and both theoretical modeling and observation-based tests of ultimate determinants of litter size have largely maintained this focus. These ecologically driven models have largely focused on the cost of post-hatch rearing of chicks. Although chick rearing (and its mammalian correlate, lactation) are demonstrably energetically intensive, this focus has generally dismissed or simply ignored the costs of egg production and incubation (equivalent in some respects to gestation in mammals) (Monaghan and Nager 1997).

Dismissing the costs of early development is almost certainly inappropriate, especially for mammals. Late gestation often confers significant costs of movement and mobility challenges for the gestating parent near term (Slonaker 1925; Dufour and Sauther 2002; Williams et al. 2016; Ladyman et al. 2018), alongside increased metabolic costs associated with the process of supporting fetal growth and storing energy in preparation for lactation. Nonetheless, the theoretical frameworks used to explain the ultimate determinants of clutch size are arguably still generally applicable to mammalian litter sizes and gestational constraints (see Risch et al. 2007; Lundblad and Conway 2021), and our intention here is simply to provide a general introduction to the theories of interest. As such, we refer to the litter size throughout this section, though it is important to remember that the original formulations of these theories tend to focus on avian reproduction and post-birth or post-hatch selection pressures.

Most of the following ultimate theoretical frameworks depend on the concept of an “optimal litter size,” and thus it is worth briefly discussing what optimal litter sizes describe before discussing the theories themselves. In short, optimal litter sizes refer to the litter size that maximizes female fitness, and theories that focus on optimal litter size assume that natural selection should act such that observed litter sizes (in free-living animals) should be approximately optimal (Lack 1954; Williams 1966). Importantly, more is not always better in an optimal litter size framework—optimal litter sizes are focused on the number of *successful* offspring individuals can produce given some environmental and evolutionary conditions. As such, at least 100 years of evolutionary biology and ecology have focused on identifying these constraints to “solve” these optimization problems and thus explain observed variation.

Three non-exclusive determinants of optimal litter size have been the focus of most theory: external resource limitations, life history trade-offs, and internal energetic ceilings. For the first two categories, physiology is not central to or deterministic for optimal litter size *per se*; instead, resource limitations and life-history strategies describe ultimate determinants of litter size. However, the ability of these ultimate selective pressures to drive changes in litter size relies upon the physiological mediators that result in individual variation. As such, natural variation in reproductive physiology provides a requisite link between ecological or environmental challenges and diversity in litter size.

### External resource limitations

External resource limitations were the first of these factors posited to explain natural variation in litter and clutch size. Lack argued that parents raised the maximum number of young allowed by food resources in their environment (Lack 1947, 1948), such that increases in food access or acquisition should permit larger clutches and litters to be raised. These arguments were largely based on Lack’s observations about latitudinal variation in clutch sizes, where clutch sizes are often larger among birds breeding at higher latitudes. Lack reasoned that the longer days at high latitudes allowed for greater time foraging, thus allowing parents to raise larger broods.

Early on, scientists recognized significant limitations to Lack’s hypothesis. For example, the food limitation hypothesis does not explain differences in clutch size among species coexisting in the same habitat and using similar food types, and it fails to distinguish between proximate causes and evolutionary origins (i.e., plasticity alone versus selection-driven traits and local adaptation) (Martin et al. 2014). Furthermore, although some small mammals show latitudinal clines in litter size (e.g., McLaren and Kirkland 1979; McLean et al. 2019; but see Ims 1997), these exemplar species are nocturnal, such that longer days at high latitudes would actually *limit* foraging time. Nonetheless, Lack’s hypothesis and its inherent limitations motivated the development of many follow-up hypotheses that propose alternative resource limitations (e.g., time) or trade-offs (e.g., activity level and predation risk) that have proven useful though not sufficiently explanatory in isolation (see Skutch 1949; Ashmole 1963; Ricklefs 1980; Martin et al. 2014; Lundblad and Conway 2021). As a result, most ecologists have landed on the idea that some combination of these ecological pressures is necessary to provide an ultimate explanation for variation in litter or clutch size (see Martin et al. 2014 and Lundblad and Conway 2021 for detailed discussions). However, the concept of external resource limitation continues to be a major mechanism for explaining variation in litter size, such that gaps between observed litter size and estimated “optimal” litter size are often attributed to failure to account for some unidentified resource limitation.

### Life history trade-offs and residual reproductive value

All Eutherian mammals are iteroparous, meaning that they attempt to reproduce multiple times across their lifetimes (but see some species in Antechinus, Braithwaite and Lee 1979; and mouse opossums, Lorini et al. 1994!). However, the likelihood of having additional reproductive opportunities is not constant across species. As a simple but extreme example, consider the difference in predation risk for an elephant versus a mouse—an elephant has a much lower predation risk and thus a greater likelihood of future reproductive opportunities, whereas a high predation risk for mice decreases the likelihood of future reproductive opportunities. Similar rationale can be applied to age—older animals will tend to have a lower chance of successfully completing another reproductive attempt relative to younger individuals. This variable risk in allocating energy toward the current reproductive attempt versus potential reproductive opportunities in the future determines an individual’s residual reproductive value (Williams 1966), which can then be used to discuss or explain patterns of reproductive investment like litter size variation.

Importantly, residual reproductive value, and thus life history strategies in the broad sense, only addresses total effort or investment per reproductive attempt. Residual reproductive value-based models do not, on their own, specify whether that reduction in effort should be expressed as a reduction in litter size to preserve effort per pup or a reduction in effort per pup within a similarly sized litter. As such, arguments about how litter size should be associated with life history strategy still usually rely on resource limitation concepts or ecological risk assessments to make quantitative statements about how litter size should change. In this way, life history strategies and residual reproductive values are an addendum to Lack’s original assertions about ecological determinants of litter size.

Within the realm of life history strategies, there are more specific “categories” of strategies that describe how effort should be variably allocated among offspring or reproductive attempts, and they usually are conditioned on environmental challenges. For example, “bet-hedging” describes strategies that are disadvantageous or suboptimal under some conditions but persist because that same strategy preserves success in challenging conditions. As such, bet-hedging strategies are often associated with less predictable environments and can result in increased variation in effort among offspring (Haaland et al. 2019). “Conservative bet-hedging,” a more specific case of bet-hedging, describes the species- or population-level strategy of consistently producing fewer, larger offspring in a litter, even though this strategy leads to lower fitness in benign environments. A conservative bet-hedging strategy is thought to arise under unpredictable environments as a function of geometric mean fitness (Einum and Fleming 2004, p. 200; Haaland et al. 2019; Okabe and Yoshimura 2022), where the conservative approach prevails in “bad” years or seasons, thereby out-competing individuals that produce larger litters in “good” years or seasons.

### Internal energetic ceilings

The final category of ultimate determinants of optimal litter size invokes energetic ceilings, suggesting that litter sizes are limited by the capacity of the gestational parent (female across mammals) to ingest, process, and transfer nutrients to developing offspring. Although lactation is generally considered the most metabolically intensive portion of reproduction for mammals, late gestation metabolic rates are also exceptionally high in association with the dramatic growth of offspring in late gestation, the increased cost of movement due to larger body size, and the necessity of preparing for lactation through organ remodeling and fat storage. For example, in deer mice, pup mass increases from an average weight of 0.3–1.8 g (a 600% increase) over the 4 days prior to birth. For an average litter size of 5, this can represent a gain of over 30% of the gestating parent’s non-pregnant mass, not accounting for maternal tissue remodeling and growth that are ongoing during this same period (Zeng et al. 2017).

For internal energetic ceilings to determine litter size, there must be fitness benefits to larger litters that animals are physiologically unable to realize. In support of this idea, estimates of optimal litter size are routinely higher than observed litter sizes in wild populations (e.g., Fleming and Rauscher 1978; Humphries and Boutin 2000; McAdam et al. 2019; Morris 1986; Wilson et al. 2009). Internal energetic limits could explain these gaps by constraining physiological capacity to support larger litters, even though it may be advantageous to do so. However, phenotypic plasticity (Leimar and McNamara 2015), “bad year” effects (Boyce and Perrins 1987), and the aforementioned “bet-hedging” strategies can all produce similar patterns of consistently lower-than-optimal litter sizes (McAdam et al. 2019). Thus, internal energetic ceilings may only explain litter size under contexts like domestication, where ecological or environmental constraints on litter size are largely removed or mitigated.

## Proximate determinants of litter size: physiology

**Fig. 1:**
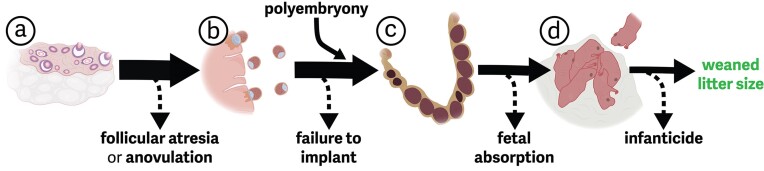
**Graphical summary of major proximate physiology that can determine litter size in mammals**. Litter size can be regulated across several developmental time points, moving from left to right in the figure. Each step is discussed in more detail in the main text. The number of follicles matured (a) often determines the maximum possible litter size, and little size reduction here can occur via follicular atresia or anovulation of mature follicles. After ovulation, (b) zygotes must implant in the endometrium, and significant loss is often associated with failure to implant. In extremely rare cases, litter size can be increased via polyembryony following implantation. (c) Fetal growth and development can be aborted throughout gestation through fetal absorption or a complete abortion. Finally, (d) post-natal infanticide can further reduce litter size prior to weaning. **Solid arrows** indicate viable offspring continuing through development, whereas **dashed arrows** indicate loss of offspring. **Dashed arrow weight** is not proportional to the expected frequency or rate. The **decreasing solid arrow weight** indicates the general rule that litter size can only be reduced across these developmental steps.

Although offspring number at birth is perhaps the most intuitive way to estimate litter size, the data from which litter sizes across mammals are derived from a large number of surrogate measures that are presumed to reflect offspring number at birth. These metrics range from the number of corpora lutea within the ovaries (a measure of the number of oocytes ovulated), blastocysts flushed from the uterus, counts of full-term fetuses, the number of offspring observed after birth (e.g., emerging from natal burrows), or placental scars. The use of any of these measures is often determined not by knowledge about relevant reproductive physiology but instead on how accessible species are across gestation and the tools available for estimation. Nonetheless, this variation reflects that there are many steps between oocyte production and birth at which litter size can be modified, often (though not always) by physiology of the gestational parent (Fig. 1). Understanding the range of potential mechanisms and the actual point of regulation can help inform how we think about the selective pressures shaping litter size evolution and the reproductive costs to the gestating parent. For example, late-gestational litter size reductions represent a different energy investment strategy than litter size reductions determined by the number of oocytes ovulated. In this section, we walk through steps of gestation—from ova maturation to birth—that influence litter size, and we highlight interesting or unique examples of variation among mammals, with our primary emphasis on Eutherians. Throughout, we have included representative figures illustrating key structures or processes involved in reproduction to provide more context to readers without a background in physiology or reproduction. Although we have endeavored to represent a generalized perspective on Eutherian female reproductive anatomy and gestational physiology, these figures are biased toward rodent and human physiology based on our own expertise. Taxon-specific variations are worth investigating for the interested reader, and several excellent texts exist that are more comprehensive in this respect (e.g., Schulkin and Power 2012; Hayssen and Orr 2017).

### Ovulation

Litter size is first controlled by the maturation of viable oocytes within the ovary. Early in follicle development and maturation, a large number of follicles develop in concert, with attrition of many of these follicles occurring as development continues. In most species, there is some point at which a number of “dominant” follicles emerge that are the fastest growing and thus become the largest follicles within the ovary (e.g., Richard et al. 2024); these dominant follicles are are destined for ovulation. As such, larger litters generally result from a larger number of oocytes proceeding to the dominant stage to be ovulated, and indeed, selection on litter size often increases ovulation rate (e.g., Durrant et al. 1980).

**Fig. 2:**
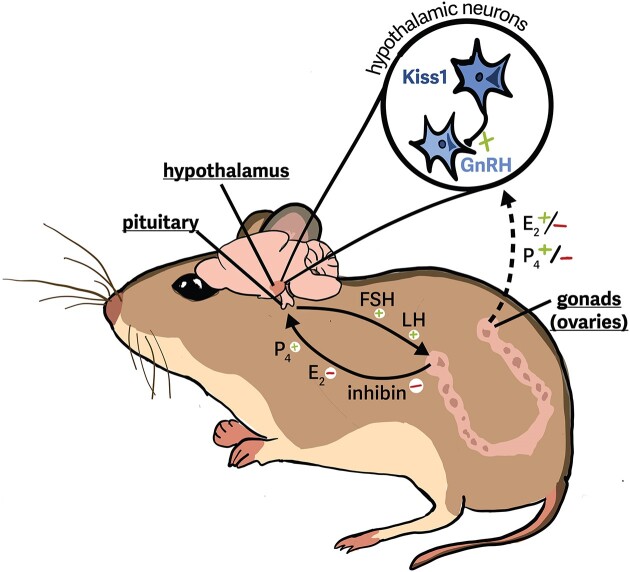
**Major signaling pathways in the hypothalamo-pituitary-gonadal (HPG) axis that influence follicular maturation, ovulation, and early pregnancy maintenance**. Follicle maturation and ovulation from the ovary are principally controlled by interactions between the **hypothalamus, pituitary**, and **ovaries** and the hormones that each produces. In the hypothalamus, Kiss1 neurons influence GnRH production and release, which in turn influences pituitary production and release of luteinizing hormone (LH) and follicle-stimulating hormone (FSH). In response to pituitary LH and FSH, specialized cells found in the ovarian follicles synthesize estradiol (E_2_) and progesterone (P_4_). These steroid hormones, in turn, feedback on to the pituitary and hypothalamus. The action of this feedback can be negative or positive, depending on the stage of the estrous or menstrual cycle (positive feedback is necessary for ovulation). Ovarian follicles also produce inhibin, which negatively feeds back into the pituitary. Solid arrows show pituitary hormone production and feedback from the ovaries. Dashed arrow shows ovarian hormone feedback on the hypothalamus. Symbols (+/-) next to hormones indicate stimulatory versus inhibitory action on the tissue.

**Fig. 3:**
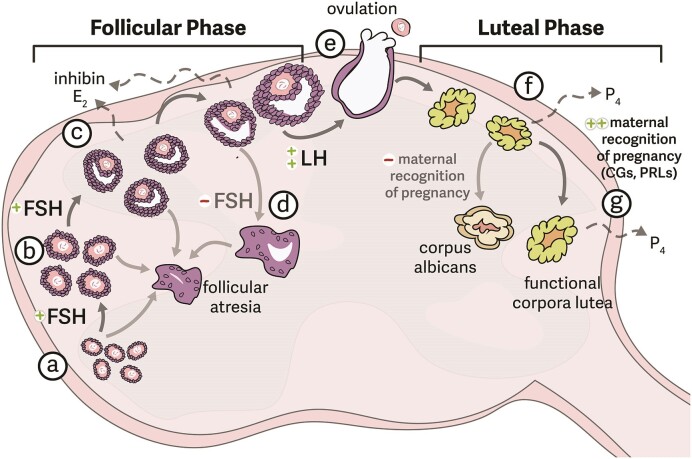
**Follicular development dynamics in the mammalian ovary**. Follicular development begins in primordial follicles (a), which are composed of the oocyte and a thin layer of granulosa cells. With the support of follicle-stimulating hormone (FSH), these follicles develop into primary (b) and then secondary (c) follicles, at which point the fluid-filled antrum begins to develop. Secondary follicles secrete increasing amounts of estradiol and inhibin, which will eventually start to suppress FSH release by the pituitary. To be “selected” to become a dominant follicle that is projected to ovulate, the follicle must develop sufficiently to become FSH-independent. Once dominant, an antral follicle become a Graffian or pre-ovulatory follicle. Throughout this process, follicles initially developing as part of the synchronous wave undergo atresia, in which they degrade and the oocyte dies (d). Mature follicles that persist despite decline FSH will be ovulated (e) in response to an estradiol-driven surge in circulating luteinizing hormone (LH), releasing an oocyte, which can then begin its journey toward the oviduct. The remnants of the ruptured follicle then become corpora lutea (f), an endocrine structure that supplies progesterone (P_4_) necessary to maintain pregnancy. However, these corpora lutea cannot persist indefinitely. Without hormones or other signals to provide maternal recognition of pregnancy (often chorionic gonadotropins [CGs] or prolatins [PRLs] coming from zygotes), these structures degrade into corpus albicans, which are usually reabsorbed by the ovary across subsequent cycles. However, in the presence of appropriate signals, the corpora lutea will remain active (g), continuing to supply the progesterone necessary to promote gestational physiology. Note that waves of follicles are nearly always being recruited from (a) primordial through (c) early secondary follicle stages, even during pregnancy, but dominant follicles that proceed through antral development are not produced. Instead, these follicles proceed through atresia (d).

Oocyte maturation and ovulation are controlled in part by top-down signaling from two hormones that are produced in and released by the pituitary gland: luteinizing hormone (LH) and follicle stimulating hormone (FSH) (Fig. 2). Divergence of the dominant follicle(s) from others in the same wave is associated with the suppression of FSH by both estradiol and inhibin secretion from the developing follicles themselves (Figs. 2 and 3; Mihm and Evans 2008). Whereas many follicles will become atretic upon the decrease in FSH, dominant follicles are able to continue development independent of FSH signaling. The mechanisms that control this “escape” among dominant follicles vary across mammals.

FSH signaling has been repeatedly associated with litter size or offspring number across mammals (Vinet et al. 2012; Mbarek et al. 2016); for example, in humans, more frequent release of FSH from the pituitary is associated with multiples in pregnancy, including dizygotic twins (Lambalk et al. 1998). However, artificial selection for large litters in lab mice has also demonstrated that LH receptor expression in the ovary can similarly promote the maturation and ovulation of an increased number of oocytes (Pomp et al. 1988). Whether LH signaling is involved in changes to oocyte maturation and ovulation more broadly, similar to FSH, will require further comparative research.

Upstream of these pituitary hormones, other neurohormones may also modulate litter size across populations or species. In goats, genetic polymorphisms in kisspeptin (KISS1) have been associated with natural variation in litter size (An et al. 2013), and the variants of the receptor for gonadotropin-releasing hormone (GnRH) have been associated with the number of follicles ovulated in pigs (Jiang et al. 2001). Other studies have identified a large number of genes that display differential expression in the follicles of pigs selected for different litter sizes, suggesting that fundamental changes to follicular growth and maturation are mitigated in part by growth pathways in follicles themselves (as opposed to top-down drivers) (Caetano et al. 2004; Fernandez-Rodriguez et al. 2011). These differentially expressed pathways within follicles appear to be involved primarily in immune regulation or function, steroidogenesis, lipid or fatty acid metabolism, and the complement cascade (Fernandez-Rodriguez et al. 2011).

In many mammals, including many ungulates and primates, litter size has evolved to a fixed litter size of one. Monovulation (ovulation of a single mature ova per cycle) generally requires reducing the number of follicles that reach a dominant stage (termed follicular deviation; Wiltbank et al. 2000; Garcia-Guerra et al. 2018). The endocrine and paracrine signaling that shape follicular development and follicular wave sizes is shared across mammals, as evidenced by the fact that supplementing or increasing FSH and/or LH availability leads to ovulation of multiple follicles across most mammals (Vinet et al. 2012). What makes monovular species unique, then, is the capacity of a single dominant follicle to produce sufficient FSH-inhibitors (largely inhibin) to suppress pituitary FSH production on its own. Elevated production of activins from the pituitary gland may increase sensitivity of the dominant follicle to FSH, allowing it to persist under lower FSH concentrations. The dominant follicle in monovular species also often exhibits a “switch” to LH-dependence (Mihm and Evans 2008).

The follicular dynamics and physiological mechanisms that lead to monovulation are largely based on work in three species: humans, domestic horses, and domestic cattle. As such, a broader exploration of the evolution of monovulation in groups like bats and marsupials (lesser studied groups where monovulation appears to have evolved independently) may provide important comparative data points in understanding how conserved or convergent the physiology of monovulation is among mammals.

### Ovulation can be disconnected from litter size

Selection on ovulatory rate can be sufficient to drive changes in litter size. However, selection experiments in a range of domestic species, including pigs, rabbits, and mice also demonstrate that increasing the number of corpora lutea and/or the number of oocytes produced often only increases resulting litter size by a few offspring, and sometimes not at all. For example, mice derived from lines selected for large litters ovulated an average of 23 ova (∼8 more than control lines); however, average litter size at birth increased to only 15 (3 more than control lines) (Eisen 1978; Pomp et al. 1988). In swine, increasing ovulated oocytes from 13 to 23 similarly only translated into a litter size increase of 2 or 3 piglets (Lamberson et al. 1991; Johnson et al. 1999). This disconnect between the number of oocytes ovulated and the number of offspring was specific to the selected line; the number of corpora lutea found in the ovaries of control line females (a proxy for ovulated follicles) corresponded fairly closely to fetus number (Lamberson et al. 1991; Johnson et al. 1999). Rabbits also show increased ovulation in response to selection on the trait, but this does not translate into increased litter sizes (Laborda et al. 2011). However, most of these selection experiments have been performed in domesticated lines likely to have been previously selected for reproductive efficiency. As such, it is difficult to determine whether the failure of litter size to increase reflects fundamental limitations to physiology to support litter size or limitation imposed by other factors that have been canalized by domestication.

Two extreme examples where ovulated oocytes and litter size are dramatically disconnected are polyovulation and polyembryony. Polyovulation involves the production of many more oocytes than will be implanted and gestated, whereas polyembryony is the formation of multiple embryos from a single zygote.

Polyovulation is relatively common across mammals, whereas polyembryony is comparatively rare. Polyovulation is found in rodents (e.g., Flamini et al. 2020), shrews, tenrecs, bats (e.g., Bueno et al. 2019), and ungulates, and it can involve the release of upwards of 800 gametes in the case of the plains viscacha (Flamini et al. 2020). Polyovulation does not appear to be used as common a mechanism for plasticity in litter size, in which animals could ovulate many ova and then control implantation or development based on environmental conditions. Most of the species that display polyovulation, including elephant shrews and plains viscacha, give birth to litter sizes that are consistently limited to only a few (< 3) offspring (Tripp 1971; Birney and Baird 1985; Sikes and Ylönen 1998; Jensen et al. 2008). The selective pressures that underlie this phenomenon remain poorly resolved. In the case of pronghorn, it may serve as a mechanism to select for the most fit offspring (Birney and Baird 1985; Sikes and Ylönen 1998)—embryos commit siblicide in utero by piercing one another with outgrowths from the chorion and allantois (O'gara 1969).

Polyembryony is best known as monozygotic twins (or any monozygotic multiple). However, up until recently, genetic correlates of polyembryony were not thought to exist, at least in humans—monozygotic embryony is still generally considered to be an unpredictable event with no heritable or predictable basis across populations or maternal age (Hamamy et al. 2004; Hoekstra et al. 2008; Smits and Monden 2011). The only well-established exception to this is found within *Dasypus* armadillos. Nine-banded armadillos invariably produce a single ovum but achieve litter sizes of four through epiblast divisions post-implantation. Polyembryony in armadillos is thought to have evolved in response to limitations in the number of sites within the uterus at which an zygote can implant (Galbreath 1985; Craig et al. 1997; Loughry et al. 1998). The genetic basis and evolutionary basis of this fixed polyembryony remains unknown though. In humans, new sequencing approaches point toward epigenetic signatures found across the ends and centers of chromosomes as distinct and shared features among monozygotic twins (van Dongen et al. 2021). It is therefore theoretically possible that, among armadillos, some genetic variation could promote similar epigenetic signatures that lead to polyembryony. Whether or not there is any overlap in the mechanisms that lead to polyembryony across mammals, more broadly, remains to be determined.

### Gamete fusion and implantation

Follicle maturation also plays a role in setting up the appropriate hormonal environment for gestation to proceed. Following ovulation, mature follicles are luteinized and form corpora lutea (CLs; see Fig. 3), an endocrine structure in the ovary that produces the steroid hormone progesterone. Progesterone is responsible for maintaining a physiological environment permissive for implantation and gestation. Non-ovulatory follicles can also be luteinized to form accessory CLs that contribute to progesterone secretion (e.g., Donaldson and Hansel 1965; Lueders et al. 2011, 2012; Cuervo-Arango and Newcombe 2013; Yanagawa et al. 2015). The number of follicles that are luteinized can impact the dosing of progestogen hormones (Hazano et al. 2021), suggesting that increases or decreases in the number of oocytes ovulated may impact early gestation success by changing the amount of progesterone secreted by CLs. In elephants, hippopotamuses, and odontocetes (toothed whales), which generally have extended gestations of singleton offspring, produce a second wave of non-ovulatory follicles that become luteinized later in gestation and produce progesterone as well, presumably to maintain the extended gestation durations (Brodie 1972; Eltringham 1999; Lueders et al. 2012). The evolutionary origins of accessory CLs are not clear.

**Fig. 4:**
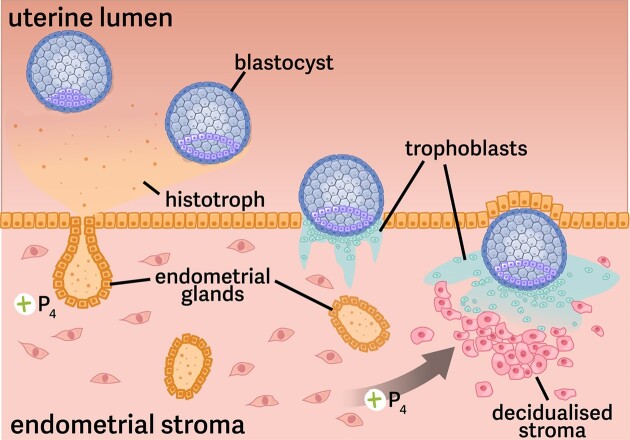
**Decidualization and implantation in the mammalian uterus**. While traveling from the oviduct to the uterus, the zygote develops into a blastocyst such that it enters the uterus ready to proceed with implantation. In response to progesterone from the ovarian corpora lutera, the endometrium lining of the uterus (bottom) has developed a larger number of endometrial glands that produce and release histotroph into the uterus as the blastocyst adheres to it. This histotrophic nutrition will serve as the primary source of energy for the developing blastocyst until the placenta is fully developed. As the blastocyst begins implantation, trophoblasts invade the endometrium and work to establish the implantation site. Interactions between these cells along with the ovarian-derived progesterone promote decidualization, or the transformation of the endometrial stroma into decidual cells. These decidual cells contribute to the development of the placenta and establish vasculature for nutrient and gas exchange for the developing fetus. Note that the processes shown are a generalized presentation of a rodent-like pregnancy. The interactions between the blastocyst and endometrium vary across mammals; see Abrahamsohn and Zorn (1993) and Enders and Carter (2006) for more..

Progesterone acts in several ways to maintain pregnancy by altering maternal physiology. One of these actions is to promote decidualization of the uterine lining, the process that prepares the endometrium in the uterus for implantation (Fig. 4). Along with estrogens derived from the endometrium itself and the ovaries, progesterone acts to promote receptivity of the endometrium to blastocyst implantation. Successful decidualization is essential for successful implantation, in which blastocysts engage with and may be engulfed by endometrial tissues. As such, successful implantation usually involves careful local regulation of the gestational parent’s immune system to prevent rejection of the embryos (Yoshinaga 2008; Fujiwara et al. 2016). Progesterone-dependent decidualization is also important for early growth of the conceptus (both prior to, during, and following implantation). Endometrial glands that develop in the uterine lining during decidualization are responsible for producing histotroph, a mixture of glycogen, glycoproteins (including mucins), lipids, and growth factors that provides a critical source of nutrition for the early conceptus prior to the functional onset of placental nutrient and gas exchange across mammals (Fig. 4; Burton et al. 2020).

The signals that mediate preparation of the uterine lining for implantation are likely to be important players in both increasing and decreasing litter size. In domestic swine, variation in litter size has been linked to allelic variants for a range of genes involved in decidualization, including steroid hormone receptors (including progesterone, Peiró et al. 2008; and estradiol, Rothschild et al. 1996; Short et al. 1997; Laliotis et al. 2017), enzymes that influence production of prostaglandins (COX2, Sironen et al. 2010), the cytokine leukemia inhibitory factor (LIF; Lin et al. 2009; Mucha et al. 2013), and pieces of the IGF/IGFBP signaling pathway (Sironen et al. 2010). In most cases, these associations between a specific allele and differential litter size appear to be breed-specific (i.e., dependent on the genetic background the allele is placed within; Peripato et al. 2004; Argente 2016). This genetic background specificity suggests that the dynamics that influence blastocyst implantation and litter size variation are almost certain to be polygenic and may depend on the combination of alleles found in both the gestating parent *and* the fetus’s genomes.

The signaling that coordinates implantation also serves to ensure that embryos are adequately spaced within the uterus—implantation sites that are not adequately spread out throughout the uterus will contribute to crowding during late gestation, which can ultimately have negative impacts on fetal growth and survival. Morphological as well as physiological factors are important in determining where and how many embryos can implant. For most mammals, the entire uterus is not receptive to implantation. In some bats, “vascular tufts” in the endometrium (local, highly vascularized regions of the endometrium) appear to limit the number of implantation sites in the uterus to one or two, perhaps serving as a critical gate on litter size in these volant mammals (Rasweiler 1992; Catalini and Fedder 2020). Uterine structure can also limit litter size by decreasing potential implantation sites. As mentioned previously in the discussion about polyembryony in armadillos, species with fused uterine horns (simplex uterus) tend to implant and successfully gestate only one or two offspring. As one would expect, then, artificial selection for larger uterine capacities can also drive increases in litter size (Spencer et al. 2012). Selection for increased litter size in tends to require increases in the length of the uterine horns (Clutter et al. 1994).

Structural constraints associated with uterine size can also be dependent on other reproductive traits like placental type. A detailed explanation about placental structure and terminology is beyond the scope of this manuscript and unlikely to be intimately linked to evolution of litter sizes; readers are encouraged to see Wildman et al. (2006), Schulkin and Power (2012), and Hayssen and Orr (2017) for more information on placental diversity in mammals. Nonetheless, for pigs, which possess a diffuse, epitheliochorial placenta, surface area for attachment between the fetal placenta and the maternal uterine tissue is directly linked to surface area for nutrient and gas exchange. This structural link means that, when holding uterine size constant, increasing litter size will decrease the average surface area per fetus that is available for exchange (and thus capacity for nutrient and gas exchange) (Ford et al. 2002). This contrasts with discoid, hemochorial placentas, where labyrinth or villous structures internal to the placenta create a much larger surface area for exchange. As such, although placental structure is not likely to direct evolution of litter size, it may influence potential to select for directional shifts in litter size in some species.

### *In utero* fetal loss

Once embryos have implanted, litter or fetal loss can be associated with dysfunction in the CL or placental capacity to maintain gestational physiology, or due to chromosomal abnormalities in the fetus and placenta. However, selective fetal resorption has also been posited as a way to defer physiological “decisions” about litter size until more information about the lactation environment is available to an individual (i.e., a mechanism for plastic modifications to litter size during late term). Late-term resorption, in which fetuses can be degraded and resorbed *in utero* without overt negative impacts to development of other offspring (e.g., Krackow 1992; Westlin et al. 1995; Giacchino et al. 2020), may be an effective way to control maternal investment during the most energy-intensive periods of reproduction (late-term growth and, more importantly, lactation).

Indeed, late-term resorptions are common in rodents, and a recent survey of domestic dogs found that they occurred in nearly 50% of canine pregnancies (14% of total implantation sites) (Lascialfari et al. 2023). Fetal loss can occur as a result of insufficient nutrient delivery or growth restriction—for example, dromedary camels twin relatively frequently, but never give birth to more than one offspring due to mid-pregnancy growth restriction and death of one of the twins (Ali 2017). In other cases, selective resorption appears to be a routine aspect of gestational physiology. For example, plains viscacha routinely implant 10–12 embryos (from over 800 ovulated), which result in only 2 viable offspring by late gestation (Flamini et al. 2020). One potential explanation may be that these additional offspring serve as “insurance policies” in the event that other oocytes are not viable (Birney and Baird 1985). One might then expect that plains viscacha (and other extreme polyovulators) have higher rates of inviable embryos relative to non-polyovulating relatives; however, to our knowledge, this has not been evaluated.

At least in mice, fetal resorption can be controlled by maternal neuroendocrine factors (Zhou et al. 2022), pointing to maternal control over the fetal resorption process. However, these neuroendocrine mechanisms were not selective (i.e., they resulted in complete resorption as opposed to resorption of specific embryos). More proximate mechanisms that could mediate selective resorptions likely involve coagulation cascades and immune interactions between maternal and fetal systems at the implantation site, which result in ischemia for an individual offspring, eventually leading to death (Clark et al. 1999). However, in general, the mechanisms that could control selective late-term resorptions are unknown.

### Post-natal litter size adjustments

Litter size may also be modified after birth through maternal infanticide, conspecific infanticide, or siblicide. In hamsters and wood rats, maternal infanticide may be selective, leading to adjustments in sex ratio to reduce investment in male offspring under challenging environments (McClure 1981; Schneider and Wade 1989; Beery and Zucker 2012). In house mice, conspecific post-partum infanticide is more common in wild colonies, where mice often breed communally (Ferrari et al. 2016, 2019). In either of these cases, litter size prior to birth may be significantly less likely to experience selection relative to maternal behaviors, and such mechanisms are likely to substantially alter the extent to which natural selection is likely to act on physiological determinants of litter size (versus maternal behavior). The extent to which maternal infanticide contributes to litter size reductions (versus complete litter loss or consumption) in other mammals is less clear, especially in rodents, where this behavior appears to be relatively, especially associated with stress. Nonetheless, the prevalence and extent to which species exhibit post-natal infanticide could provide important context for understanding how mechanisms that control *in utero* litter size are subject to selection pressure.

In contrast to maternal and conspecific infanticide, siblicide is relatively uncommon in mammals. Spotted hyenas are arguably the only mammal where siblicide is known to be a major determinant of the total maternal cost of reproduction (Smale et al. 1999; Hofer and East 2008). In spotted hyenas, siblicide has been hypothesized to have arisen in response to low or unpredictable food environments. The fact that siblicide is otherwise extremely uncommon in mammals (but relatively common in some avian species; Mock 1984; Mock et al. 1990) suggests that there is more to learn about the selective forces that promote or inhibit siblicide as a mechanism for moderating maternal reproductive investments across offspring within a litter.

### Plasticity in litter size

Plasticity, or the capacity of an individual to vary litter size, also contributes to variation in litter size across species, and the ability to display plasticity or the scope of that response may also evolve in response to natural selection.

Perhaps the most predictable source of plasticity in litter size arises across parities, where first litters are almost always smaller than subsequent litters. Parity-dependent increases appear to be a requisite step toward achieving large litters: sheep selected for larger litters progressively increase in fertility up to 6 years of age, whereas low or control lines are consistent across parities (Schoenian and Burfening 1990). In line with this pattern, the degree to which parity increases litter size in *Peromyscus* species appears to vary with average total litter size (Wilsterman and Cunningham 2022). Many of the allelic variants found in pigs that impact litter size are parity-dependent, either impacting first parity or later parities, but not both (Rothschild et al. 1996; Short et al. 1997; Sironen et al. 2010). These patterns suggest that the first parity has some “programming” effects on maternal physiology to acclimate to subsequent pregnancies, but shockingly little is known about the physiological mechanisms that influence parity-dependent plasticity, though it is nearly universal.

Seasonal plasticity in litter size has also been documented in some species. Late-season decreases in litter size may be an adaptive response to the fact that late-season litters (and their mothers) have less time to prepare for up-coming inhospitable seasons. Reductions in litter size may allow the gestational parent to invest more per offspring. Alternatively, smaller litters “cost” less in total but still may afford some fitness benefit if offspring manage to survive. Most evidence for seasonal plasticity is derived from population-level studies, where individuals are not tracked across a season. For example, house mice appear to produce progressively smaller litters across the breeding season (i.e., litter size peaks in the spring) (Singleton et al. 2001). However, seasonal patterns in litter size that are observed at a population level may be a function of parity-dependent plasticity in litter size and demographic changes, where late-season reductions in litter size are the result of first-time reproductive attempts by young-of-the-year females, which produce smaller litters. Tracking individuals and controlling for body size (as a reasonable proxy of age) are critical to disentangling these effects.

Physiological plasticity can generate increases (i.e., Tannerfeldt and Angerbjörn 1998; Boutin et al. 2006) or decreases (e.g., Gosling 1986; Pratt and Lisk 1989; Krackow 1992) in litter size in response to environmental or social cues. The mechanisms that mediate physiological plasticity remain largely unresolved. For example, we are not aware of any studies that have resolved whether increases in litter size in response to anticipated food availability, as is well-documented in North American red squirrels (e.g., McAdam et al. 2019; Dantzer et al. 2020; Petrullo et al. 2023), are a function of differential ovulation, implantation, or the suppression of resorptions. Given that uterine capacity tends to covary with maximum litter sizes (see earlier sections), we might expect evolution to act on resorption and suppression thereof to enable animals to modulate litter size while maintaining the structural capacity to support larger litters when it is advantageous. The placenta may play a critical role in mediating this type of plasticity based on its role in controlling maternal/fetal interactions (for more, see Drews et al. 2020; Bowman et al. 2021).

Partial and complete litter resorptions are often observed in response to social and environmental stressors, and they may be associated with maternal body composition or lipid stores. The extent to which partial resorptions can be adaptively “selective” across such examples remains unclear, in large part because the mechanisms are not well defined. Differentiating between adaptive fetal loss or resorption and non-adaptive or maladaptive loss is nontrivial, and invoking adaptationist explanations for late-term resorptions should be argued with careful attention to potential mechanisms as well as the cost/benefits to the gestating parent.

### Co-evolution between litter size and other traits

Litter size does not evolve in isolation. We have already discussed this idea with regards to structural constraints (or opportunities) in the uterus. However, associations between litter size and other aspects of organismal biology extend well beyond the uterus. A full treatment of the co-evolution of litter size with other traits is beyond the scope of this manuscript; however, we highlight a few important examples here.

In general, selection studies show that female body weight and litter size maintain a positive correlation; body size or weight increases along with litter size within a lineage (e.g., Pomp et al. 1988). Increasing body size first provides structural space, particularly in the uterus, for enlarged litters, but the large mass of the gestating parent may also allow them to maintain the capacity to support growth of offspring *in utero* through larger body fat stores and increased processing capacity of the liver, gut, and other organs. This structural component is consistent with the dramatic expansion of maternal organs, including the liver, gut, and spleen, that develop during early pregnancy in anticipation of late gestation and lactation-related energy expenditures (Bustamante et al. 2008, 2010; Dai et al. 2011). In mice, average pup mass is stable in lines selected for larger litters (in which maternal body size also increased); however, within-litter variation in pup mass increased (Bakker et al. 1978; van Engelen et al. 1995), which is likely to impact survival likelihood of offspring post-weaning unless the gestating parent can effectively control lactation effort across the litter. The extent to which these structural correlations are responsive to or drivers of shifts in life history (i.e., relatively “fast” versus “slow” life histories) within lineages could be an interesting area for future work.

Of course, *across* mammals, these correlations tend to reverse. For example, larger-bodied mammals tend to have *fewer* young when compared with lineages of smaller-bodied mammals (Hayssen and Orr 2017). The larger set of traits that co-evolve *across* species, including traits like gestation duration and age at first reproduction (reviewed nicely in Bielby et al. 2007), are often not reflected by *within* species correlations. Correlated sets of reproductive traits across species can generally be organized into two sets of traits: those that are associated with the trade-off between offspring number and size (gestation length, neonatal body mass, and litter size) and those that are associated with the timing of reproduction (age at sexual maturity, interbirth interval, and weaning age [lactation duration]) (Bielby et al. 2007). Identifying clades of species that show larger variation *within* either of these categories is likely to be particularly useful for understanding how and when these correlated sets of traits evolve. On the other hand, lineages that lack variation in some traits (like litter size) but vary in other components of these correlated traits (like neonatal body mass or gestation length) can offer complementary insight into how evolutionary physiology shapes trait diversification. For example, artiodactyls and primates lack variation in litter size, largely producing a single offspring per reproductive attempt, but they still exhibit variation in gestation duration and neonatal body mass. Contrasting examples of directionality of litter size evolution within clades may also be a fruitful area for further study. Within artiodactyls, pigs and peccaries (*Suidae*) display considerable variation in litter size: whereas *Sus* species tend to produce >6 offspring per litter, most other *Suidae* family members only produce anywhere from 2 to 4 offspring (Sutherland-Smith 2015). This variation within a clade that is otherwise invariant with 1 or 2 offspring may provide an opportunity to understand how large litter sizes arise on an evolutionary background of relatively smaller litters. In contrast, rodents naturally offer a useful comparative system for understanding mechanisms by which small and more precocial litters arise from lineages that produce large litters comprised of relatively altricial young.

There are also a number of traits that are linked to reproductive physiology but are comparatively poorly incorporated in the life history literature and thus deserve further attention. As an example, whether species rely more on stored energy to fuel reproduction versus active consumption (i.e., capital versus income breeding strategies; Houston et al. 2007; Stephens et al. 2009; Williams et al. 2017) has not been widely incorporated into broader discussions about evolution of reproductive traits in mammals. The relative bias toward studying income-breeding species is most noticeable in the context of plasticity in reproductive traits (as discussed in Lane et al. 2019), where the capacity for species to manage capital versus income resources is likely to also impact their capacity to modulate reproductive investment under variable environmental conditions.

Finally, it is worth acknowledging that most of the discussion here has focused on litters of multiples in Eutherian mammals. In doing so, we have made some exclusions that deserve further attention, especially when considering how reproductive traits shape lineage diversification and resilience. First, we have paid relatively little attention to the preponderance of singleton litters found across mammals. Singleton litters require unique ovarian physiology (as is briefly discussed above), and the evolution of singleton litters may impact the signals that control timing of parturition and lactational physiology (including relative energy investment pre- versus post-natally) (Hayssen and Orr 2017). As such, evolutionary physiology of singleton litters is likely to yield unique and important insights relevant to Eutherian life history. Second, we have largely excluded marsupials from our discussion. Marsupials are considered lactation specialists, in contrast to the Eutherian specialization on gestation and placentation. We expect these differences between Eutherians and marsupials to shape how reproductive physiology has evolved in either lineage. Comparative work on the extent to which physiology, life history, and reproductive traits have co-evolved in either group is thus likely to lead to interesting insights about the extent to which fundamental trade-offs underlie mammalian evolution.

### Conclusions

Currently, we know almost nothing about the physiology that underlies natural variation in litter size across Eutherian mammal diversity. The vast majority of genotype-to-phenotype maps explaining determinants of litter size are derived from studying domesticated lines, and they largely lack a proven physiological mechanism or process that links genotype and phenotype. Although there is ample evidence that reproductive traits like litter size and offspring conditions can be selected on in either direction (even in relatively inbred mouse strains), we have no evidence that the loci under selection in the lab also explain variation in these traits in wild populations. The relative roles of plasticity and local adaptation in these traits in the wild will be critical for identifying and understanding how genetic mechanisms translate into phenotypic variation. At the same time, although we do know a lot about the physiology that controls reproductive biology, genomic scans have revealed relationships between litter size and genes that have no well-described function directly mediating reproductive physiologies that shape litter size (e.g., many of the genes identified in Tao et al. 2021). Organismal, experimental study of reproductive physiology will continue to be an essential piece to understanding the importance of the many molecular candidates that are emerging from genomic scanning methods.

The ecological context in which we expect litter size to evolve is also important to consider, especially with regards to the evolution of plastic versus fixed litter sizes. The extent to which these environmental factors may select for variation *within* a litter (as opposed to directional selection on pup number) will also provide significant context for how we look for and experimentally investigate litter size variation. Finally, unpredictable or low-probability events are likely to play important roles in ecologically driven selection on reproductive traits like litter size. For example, the unlikely event of successful recruits from a late-season, second annual litter remains extremely influential in species like the North American red squirrels because each additional offspring has such a high impact on total fitness (Humphries and Boutin 2000; Boutin et al. 2006; McAdam et al. 2019; Williams et al. 2014). In other contexts, common but unpredictable events like nest predation may significantly obscure any selection on litter size traits (e.g., Morris 1986, 1992; Morris et al. 2011).

Box 2: **What research areas will benefit from a mechanistic understanding of the evolution of litter size?**

(1) **Life history evolution—**Are there mechanisms underlying litter size and co-evolving traits that are shared with life history variation in other vertebrates or are some trait linkages unique to Eutherian mammals? Are changes in litter size across a species or clade’s history gradual or rapid?

(2) **Evolutionary physiology**—Are there physiological mechanisms that underlie co-evolution of litter size with other traits? Which of these scale within and among species? When or how often are the same processes coopted to produce a given outcome (here, increasing or decreasing litter size—singleton versus multiple litters etc.)?

(3) **Evolutionary processes—**To what extent do physiologic (energetic) constraints versus ecological pressures (predation risk, variable environments) limit or direct evolution of litter sizes in natural environments?

(4) **Genetics and genome evolution**—What is the basis of epistasis in litter size evolution? To what extent does epistasis play a role in limiting evolution of litter size in natural populations, particularly in those where we know inbreeding is relatively common?

Pursuing the proximate (physiological) mechanisms that explain variation in litter size is likely to have considerable pay-off. Developing a mechanistic understanding of the evolution of litter size will inform how we think about life history strategies and their evolution, evolutionary physiology, evolutionary biology more generally, and genetics and genomics (Box 2). As a field, evolutionary physiology is currently well-positioned to pursue these questions by leveraging current tools from high-throughput genomics and large observational datasets (e.g., Colella et al. 2021). Nonetheless, collaborative science that brings organismal and experimental physiology together with evolutionary theory and genetics/genomics has the most potential to yield transformative discoveries about mammalian evolution and diversity in the coming decades. Most important among these, though, will continue to be organismal biology and physiology, which are the required link between an individual’s environment and the most critical determinant of their fitness: reproduction.

## Data Availability

No new data were generated or analyzed in support of this research.

## Notes

From the symposium “What do trade-offs mean to reproducing females?: An integrative look at whole-organism trade-offs” presented at the virtual annual meeting of the Society for Integrative and Comparative Biology, January 2-6, 2024.
